# Non-fungible token integration in neurosurgery: a technical review

**DOI:** 10.1007/s10143-023-02119-9

**Published:** 2023-08-22

**Authors:** Aaron Lawson McLean

**Affiliations:** Department of Neurosurgery, Jena University Hospital – Friedrich Schiller University Jena, Am Klinikum 1, 07747 Jena, Germany

## Introduction

In the rapidly evolving world of digital technology, few concepts have garnered as much attention and intrigue as non-fungible tokens (NFTs). Originating from the domain of art and collectibles, NFTs have transcended their initial use cases to impact various fields, from entertainment to real estate, and now, healthcare [1]. While fields like ophthalmology, dermatology, and plastic surgery have begun exploring the potential applications of NFTs, neurosurgery stands at the threshold of a new era, where digital ownership and blockchain can revolutionize patient care, data management, and surgical innovations [2–5].

Neurosurgery, with its intricate procedures and reliance on detailed imaging and data, offers a fertile ground for NFT applications [6]. Whether it’s ensuring the authenticity of patient scans, safeguarding the intellectual property of novel surgical techniques, or even creating a transparent ledger for pharmaceutical transactions, NFTs hold promise to bring about enhanced security, autonomy, and innovation to the realm of neurosurgery. As technological advancements intertwine with medical progress, the importance of integrating secure and transparent digital systems becomes paramount. In this context, this review delves into the nuances of NFTs, elucidating their potential roles and impacts within the multifaceted domain of neurosurgery.

For a clearer understanding of the specialized terminology used in this paper, readers are encouraged to refer to the glossary of key terms presented in Table 1.

**Table 1:** Glossary of key terms in blockchain and NFTs

Term	Description
Blockchain	A decentralized ledger of all transactions across a network
Cryptography	A method of protecting information by transforming it into an unreadable format
Decentralization	The distribution of functions and powers away from a central authority
Immutable	Unchanging over time or unable to be changed
Metadata	Data that provides information about other data
Minting	The process of creating a new NFT
Non-fungible token (NFT)	A type of digital asset representing ownership of a unique item or piece of content, usually stored on a blockchain
Proof-of-stake	An alternative to proof-of-work, where participants prove ownership of a certain amount of cryptocurrency
Proof-of-work	A consensus algorithm used in blockchain where participants solve complex mathematical problems
Public/private key	A cryptographic system that uses pairs of keys: public keys (known to everyone) and private keys (kept secret)
Smart contract	Self-executing contracts with the terms of the agreement directly written into code
Tokenize	The process of converting rights to an asset into a unique digital representation on a blockchain

## Methodology

The author (ALM) conducted a comprehensive scoping review to explore the potential applications of NFTs in neurosurgery. A systematic search was carried out using key electronic databases: PubMed, Embase, IEEE Xplore, and Google Scholar, without date restrictions. The search was executed in August 2023. To ensure a broad capture of relevant literature, the search terms utilized were “(“non-fungible token*” OR NFT OR NFTs OR blockchain OR “block chain”) AND (neurosurg* OR “neurological surgery”).” Given the novelty of the topic, grey literature sources, including preprint repositories and conference proceedings, were also scrutinized to capture emerging research and concepts in the field. Articles not written in English, those that were merely abstracts, and case reports were excluded to maintain the specificity of the review. Articles that specifically discussed the applications, challenges, or innovations of NFTs within neurosurgical practices or related patient data management were included. Further pertinent articles were identified and sourced from the reference lists of the initial set of articles. Additionally, relevant texts from non-neurosurgical medical domains and ancillary fields were examined to gain a comprehensive understanding and to identify potential cross-disciplinary applications and insights. Data from these articles were extracted and synthesized to inform the main content of the review.

## Blockchain and NFTs: a primer

At the foundation of NFT technology is the blockchain — a decentralized ledger system that supports cryptocurrencies and decentralized applications. But what exactly is a blockchain? In essence, a blockchain is a chain of blocks, where each block contains data, and each subsequent block carries a unique cryptographic signature, ensuring the integrity and immutability of the data stored within [7–9].

NFTs are unique tokens minted on blockchains. Unlike cryptocurrencies like Bitcoin or Ethereum, which are fungible and interchangeable, NFTs are distinct and cannot be exchanged on a one-to-one basis [10]. This uniqueness is what grants NFTs their name — non-fungible. When an NFT is minted, it’s associated with a specific piece of digital content, be it an artwork, music, or even medical data. The metadata of this content, akin to a certificate of authenticity, is recorded on the blockchain. This ensures proof of ownership and provides a transparent trail of any transactions or changes made to the associated content. While the most popular platform for NFTs is currently the Ethereum blockchain, various other blockchains also support NFTs, each bringing its own set of features and benefits [11].

For a detailed, sequential overview of how blockchain technology and NFT function, especially in the context of medical data management, refer to Table 2.

**Table 2:** Blockchain and non-fungible tokens (NFTs) — a sequential overview. This table provides a concise step-by-step breakdown of how blockchain and NFT function, especially in the context of medical data

Step	Description
1. Data creation	Medical data, be it imaging, genomic information, or patient records, is generated
2. Digital tokenization	This data is represented digitally, ready to be linked to a unique token
3. Blockchain initialization	A digital ledger, known as a blockchain, is set up to record transactions. This can be on existing platforms like Ethereum or newer healthcare-specific chains
4. NFT minting	A unique non-fungible token (NFT) is created (“minted”) to represent the specific piece of data. This process gives the data a unique identifier on the blockchain
5. NFT ownership	Once minted, the NFT is assigned to an owner, usually the patient or the institution that created the data
6. Data verification	Anytime the data is accessed or transferred, the blockchain verifies the authenticity and ownership using the NFT
7. Data sharing/selling	The owner can choose to share or sell the data. If sold, the NFT’s ownership can be transferred to the new owner
8. Royalties and resale	If the NFT is set up with royalties, any future sales or transfers ensure the original owner receives a percentage of the transaction
9. Data access control	The owner can grant or revoke access permissions to the data linked to the NFT
10. NFT destruction	If needed, NFTs can be “burned” or destroyed, removing their representation from the blockchain

## Potential applications of NFTs in neurosurgery

NFTs, with their unique ability to authenticate and provide undisputed digital ownership, can offer transformative solutions to longstanding issues in data management, procedure verification, and patient engagement [12, 13]. This section delves into specific applications where NFTs might play a pivotal role in reshaping neurosurgical practices, enhancing both patient care and operational efficiency.

### Patient data ownership and control

In neurosurgery, the precision and clarity of diagnostic imaging are paramount [14]. Procedures often rely on detailed MRI and CT scans. NFTs can provide a framework for patients to have indisputable ownership of their diagnostic images. This ownership isn’t just about possession; it’s about control. Patients can choose to share their scans with specific medical professionals, research entities, or even for broader educational purposes. This controlled sharing ensures patient privacy while still allowing for the potential aggregation of data for larger studies or machine learning applications.

### Genomic data in neurological disorders

Neurological conditions often have underlying genetic components. As genomic sequencing becomes more commonplace, there’s an increasing amount of data about individual genetic predispositions to certain neurological conditions or even the genetic constitution of individual tumors [15]. NFTs can serve as a tool for individuals to control and share their genomic data [16]. While the primary purpose might be for personalized treatment plans, there’s also the potential for individuals to contribute their data to broader research initiatives, aiding in the development of treatments or understanding disease progression.

### Enhancing clinical trial record security

Clinical trials are the cornerstone of medical advancements, and the integrity of their records is paramount [17]. Neurosurgical trials, given their complexity, generate a vast array of data points, ranging from patient demographics to intricate procedural outcomes [18]. Ensuring the authenticity, tamper-proof nature, and traceability of this data is essential [19]. NFTs can be employed to tokenize individual trial records, granting them a unique digital identity on the blockchain [20, 21]. This ensures that each trial entry is original and hasn’t been altered post-registration. Moreover, the transparent nature of the blockchain ensures a verifiable trail of any data changes or access, instilling confidence in both participants and regulatory bodies. By leveraging NFTs, neurosurgical clinical trials can achieve enhanced data security, minimize fraudulent activities, and uphold the highest standards of scientific research.

### Verification of neurosurgical procedures and achievements

The field of neurosurgery is constantly evolving, with new techniques and procedures being developed regularly [22]. NFTs can be employed to validate the introduction and successful implementation of these novel methods. Surgeons could use NFTs as a form of credentialing for specific procedures, ensuring that they’ve undergone the necessary training and have achieved a certain level of proficiency. Furthermore, institutions can issue NFT-based verifications for completed training programs, workshops, or milestones achieved by neurosurgeons [23].

### Pharmaceutical implications

Neurosurgical treatments often involve the use of specialized medications, for example, 5-aminolevulinic acid hydrochloride (Gliolan) for tumor resections, selumetinib (Koselugo) for neurofibromatosis type 1–related inoperable plexiform neurofibroma, or bevacizumab (Avastin) for neurofibromatosis type 2–related vestibular schwannomas [24–26]. Ensuring the authenticity of these medications is crucial. NFTs can serve as a verification tool, ensuring that the drugs used are genuine and have passed all necessary quality controls [27]. By integrating NFTs into the pharmaceutical supply chain, it’s possible to track the journey of a drug from the manufacturer to the patient, ensuring transparency and reducing the risk of counterfeit medications entering the system [28].

### Tokenized assurance for refurbished medical devices

Refurbished medical devices present economic advantages, yet their reintroduction to the healthcare system often sparks concerns about quality and potential counterfeit risks [29, 30]. An NFT-based mechanism can address these challenges by providing a transparent and verifiable record of each device’s refurbishment journey. By employing NFTs, each stage of refurbishment can be documented, thereby bolstering confidence in the device’s authenticity and safety [31]. Such a system may not only deter fraudulent activities but can also enhance the trustworthiness of these vital medical tools within neurosurgery.

### Virtual reality and augmented reality in neurosurgery

Virtual reality (VR) and augmented reality (AR) are becoming integral in surgical planning and patient education. Surgeons can use VR to simulate complex procedures, while patients can use AR to better understand their conditions and treatments [32, 33]. NFTs can be utilized to authenticate these VR and AR models, ensuring they are based on accurate data and have been developed by certified professionals [34]. Such authentication may be vital in maintaining the trust and reliability of these digital tools.

## Monetization models for NFTs in neurosurgery

NFTs, celebrated for their unique value proposition around digital ownership and authenticity, introduce a myriad of monetization possibilities in neurosurgery [35]. It’s crucial to clarify that the following exploration of potential monetization models is presented for consideration and discussion, and not as an endorsement. While patient care and ethical considerations remain paramount, it’s beneficial to be aware of the diverse ways NFTs might be integrated into the economic fabric of neurosurgery.

### Patient-driven sales of medical data

In the current era of data-driven medical research, there’s an increasing interest in acquiring expansive datasets [36]. With NFTs solidifying patients’ unequivocal ownership of their medical data, individuals have the unique opportunity to monetize their anonymized records for research endeavors [37, 38]. Such a model necessitates a comprehensive and transparent consent process, ensuring patients are fully aware of the ramifications and potential benefits of their choices [16, 39].

### Licensing of surgical techniques and innovations

Innovative surgical techniques or proprietary surgical tools could be tokenized and licensed to other professionals or institutions. Instead of a one-time sale, neurosurgeons or institutions could charge a recurring fee for the continued use of their innovations.

### Educational content, scientific manuscripts, and virtual training modules

Neurosurgeons could create and tokenize detailed training modules, virtual surgeries, case studies, scientific manuscripts, and their supporting datasets. By doing so, they not only ensure the authenticity and originality of their work but also pave the way for a new era of digital dissemination and access. Medical students, institutions, researchers, or other professionals could purchase access to these educational and research-based NFTs, ensuring genuine content while simultaneously providing a potential revenue stream for the creators.

### Premium patient services

In a more patient-centric approach, neurosurgical clinics could offer tokenized premium services. Patients holding a particular NFT could receive benefits such as priority scheduling, access to detailed digital reports, or virtual consultations.

As an example, NFTs could be harnessed to facilitate access to specialized medical services, such as outpatient rehabilitation. Consider a patient who requires a tailored rehabilitation program following a complex neurosurgical procedure. Instead of traditional referral systems or insurance approvals, healthcare providers or insurers could issue NFTs that represent a package of specific rehabilitation services. Upon acquisition, patients could redeem these NFTs at partnered outpatient centers, ensuring they receive the exact suite of services tailored to their recovery needs. Not only could this streamline the patient’s access to care, but it also offers a transparent and verifiable method of ensuring service quality and authenticity. Furthermore, the decentralization aspect of blockchain, upon which NFTs are built, can empower patients by giving them direct control over their rehabilitation journey. They can choose when and where to redeem their NFT and even transfer or sell it should they decide on an alternative rehabilitation pathway.

### Collaborative research grants and funding

Tokenized research projects could attract funding from institutions, pharmaceutical companies, or even public grants. By purchasing an NFT associated with a research project, funders could receive periodic updates, early access to findings, or even acknowledgment in publications.

### Royalties from resales

One of the intrinsic features of NFTs is the ability to embed royalties [40]. Every time an NFT is resold, the original creator can receive a percentage of the sale. This ensures that neurosurgeons or institutions continue to benefit from the increasing value of their tokenized assets.

## Challenges and limitations

While the integration of NFTs into neurosurgery offers promising avenues, it’s essential to approach this frontier with a balanced perspective. The implementation of any new technology, especially one as disruptive as NFTs, brings with it inherent challenges and potential limitations [7, 41]. From technical hurdles to ethical dilemmas, these challenges warrant careful consideration to ensure that the adoption of NFTs aligns with the overarching goals of patient safety, data security, and ethical medical practice [42].

### Energy and environmental concerns

One of the primary criticisms of blockchain technology, and by extension NFTs, is the energy consumption associated with maintaining and validating the blockchain, especially for proof-of-work systems [43]. This energy-intensive process has raised environmental concerns, especially given the carbon footprint of large-scale mining operations. While strides are being made in transitioning to more energy-efficient consensus mechanisms, such as proof-of-stake, the environmental impact remains a significant point of contention [44].

### Data security and privacy implications

Though blockchain is praised for its security and immutability, it’s essential to differentiate between the security of the transaction history and the data to which an NFT might link [45]. If an NFT points to data stored on a centralized server, that data remains vulnerable to breaches or hacks. Furthermore, while the blockchain can verify the authenticity of an NFT, it cannot guarantee the accuracy of the associated data [46]. For neurosurgical applications, where accuracy is paramount, this is a significant consideration.

### Ethical considerations

The ability to tokenize medical data, procedures, and even genomic information introduces a host of ethical questions [16]. If patients can monetize their medical data, it could lead to potential exploitation, especially in populations that might be financially motivated to share sensitive information [47]. Additionally, the idea of tokenizing surgical techniques or procedures could limit the free exchange of knowledge in the medical community, potentially hindering collaborative advancements.

### Technical barriers and usability

While the concept of NFTs and blockchain might be familiar to tech-savvy individuals, it remains a complex topic for many. For widespread adoption in the neurosurgical community, there’s a need for user-friendly interfaces and platforms that abstract away the technical complexities while retaining the benefits of the technology. Training and education will also play a pivotal role in bridging this gap.

### Regulatory and legal challenges

The integration of NFTs into the medical field will undoubtedly face regulatory scrutiny [48]. Governments and regulatory bodies will need to establish frameworks for the use, exchange, and sale of medical data or tokenized procedures [49]. These legal challenges could slow adoption and introduce additional complexities for practitioners wishing to leverage the technology.

## Future prospects and research directions

The path ahead requires targeted research to validate applications, refine methodologies, and ensure optimal integration. The upcoming segments detail specific areas of interest and potential research directions that can shape the future role of NFTs in neurosurgery.

### Optimizing blockchain technology for healthcare

The traditional proof-of-work blockchain model, with its high energy consumption, might not be sustainable for widespread healthcare applications. However, emerging consensus mechanisms, such as proof-of-stake and federated blockchains, offer more energy-efficient alternatives. Research into making these models compliant with healthcare requirements can open doors for more sustainable NFT use in neurosurgery.

### Developing secure platforms for medical data exchange

As the digitization of medical records and imaging continues to grow, there’s a clear need for secure platforms that allow for easy exchange of this data [50]. Integrating NFTs into these platforms can provide added layers of authentication and ownership. Research can focus on creating user-friendly platforms that harness the power of NFTs without overwhelming users with technical details.

### Ethical and patient-centric approaches to data monetization

While monetizing medical data has its pitfalls, it also offers opportunities. Future research can focus on developing models that prioritize patient welfare, ensuring that patients understand the implications of sharing their data and are fairly compensated for it. Ethical guidelines and best practices can be formulated to guide the process.

### Collaborations between tech developers and neurosurgeons

For NFTs to be effectively integrated into neurosurgery, collaborations between blockchain experts, software developers, and neurosurgeons are crucial. Such collaborations can lead to tools and platforms specifically tailored for neurosurgical applications, ensuring that the technology meets the unique needs of the field.

### Regulatory frameworks and standardization

With the growing interest in NFTs in healthcare, there’s a clear need for standardized protocols and regulatory frameworks [13]. Research can be directed towards understanding the implications of NFTs in healthcare settings and developing guidelines that ensure patient safety, data privacy, and compliance with existing medical laws.

### Exploring NFTs in medical education and training

Beyond patient data and procedures, there’s potential for NFTs to play a role in medical education. Tokenized modules, virtual surgeries, or even patient cases can be used for training purposes, ensuring that students and trainees are accessing authentic and approved educational material.

## Conclusion

The integration of NFTs into neurosurgery presents a blend of exciting opportunities and notable challenges. As with any technological advancement in healthcare, the primary focus remains the betterment of patient care, ensuring safety, authenticity, and precision in treatments and interventions.

NFTs have the potential to reshape the way neurosurgeons interact with patient data, authenticate procedures, and even contribute to research and education. Their capacity to offer undisputed ownership and control over digital assets can redefine data sharing, research collaborations, and even the monetization of specific medical assets. However, it’s imperative to navigate the ethical, environmental, and technical challenges associated with this technology.

While the current state of NFTs in neurosurgery is nascent, the trajectory suggests a growing interest and potential for wider adoption in the coming years [42]. Collaborative efforts between technologists, neurosurgeons, and regulatory bodies will be crucial in ensuring that NFTs find a place in neurosurgery that is both innovative and ethically sound. As the field continues to evolve, it remains crucial for the neurosurgical community to stay informed, engaged, and proactive in shaping the direction of NFT integration, ensuring that it aligns with the core values and goals of the profession.

## Data Availability

All data pertinent to this study are encompassed within the article. No supplementary data were generated or analyzed during the creation of this paper.

## References

[1] Russell F (2022) NFTs and value. M/C Journal 25. 10.5204/mcj.2863

[2] Bekisz JM, Boyd CJ, Daar DA, Bass JL (2022). Medicine as art: the potential role of nonfungible tokens in plastic surgery. Plast Reconstr Surg.

[3] Tian WM, Blau JA, Rames JD, Hollenbeck ST (2022). Nonfungible tokens in plastic surgery. Plast Reconst Surg Global Open.

[4] Pietris J, Bacchi S, Wiech S, Tan Y, Kovoor J, Gupta A, Casson R, Chan W (2023). Non-fungible tokens in ophthalmology: what is it good for?. Eye.

[5] du Crest D, Tequi C, Dessi F (2023). Are non-fungible tokens the barrier to realizing the Dermoverse?. J Eur Acad Dermatol Venereol.

[6] Mofatteh M (2021). Neurosurgery and artificial intelligence. AIMS Neurosci.

[7] Belk R, Humayun M, Brouard M (2022). Money, possessions, and ownership in the metaverse: NFTs, cryptocurrencies, Web3 and Wild Markets. J Bus Res.

[8] Gad AG, Mosa DT, Abualigah L, Abohany AA (2022). Emerging trends in blockchain technology and applications: a review and outlook. J King Saud Univ - Comput Inf Sci.

[9] Huynh-The T, Gadekallu TR, Wang W, Yenduri G, Ranaweera P, Pham Q-V, da Costa DB, Liyanage M (2023). Blockchain for the metaverse: a review. Futur Gener Comput Syst.

[10] Ante L (2022). The non-fungible token (NFT) market and its relationship with Bitcoin and Ethereum. FinTech.

[11] Ko K, Jeong T, Woo J, Hong JWK (2023) Survey on blockchain-based non-fungible tokens: history, technologies, standards, and open challenges. Int J Netw Manag. 10.1002/nem.2245

[12] Kostick-Quenet K, Mandl KD, Minssen T, Cohen IG, Gasser U, Kohane I, McGuire AL (2022). How NFTs could transform health information exchange. Science.

[13] Musamih A, Salah K, Jayaraman R, Yaqoob I, Puthal D, Ellahham S (2023). NFTs in healthcare: vision, opportunities, and challenges. IEEE Consum Electron Mag.

[14] Boekestijn I, van Oosterom MN, Dell’Oglio P, van Velden FHP, Pool M, Maurer T, Rietbergen DDD, Buckle T, van Leeuwen FWB (2022). The current status and future prospects for molecular imaging-guided precision surgery. Cancer Imaging.

[15] Phillips KA, Trosman JR, Kelley RK, Pletcher MJ, Douglas MP, Weldon CB (2014). Genomic sequencing: assessing the health care system, policy, and big-data implications. Health Aff.

[16] Zarchi G, Sherman M, Gady O, Herzig T, Idan Z, Greenbaum D (2023). Blockchains as a means to promote privacy protecting, access availing, incentive increasing, ELSI lessening DNA databases. Front Digit Health.

[17] Loder E, Godlee F, Barbour V, Winker M (2013). Restoring the integrity of the clinical trial evidence base. Bmj.

[18] Suki D, Wildrick DM, Sawaya R (2018). A time-tested information system in neurosurgical oncology. Front Oncol.

[19] Benchoufi M, Altman D, Ravaud P (2019). From clinical trials to highly trustable clinical trials: blockchain in clinical trials, a game changer for improving transparency?. Front Blockchain.

[20] Wong DR, Bhattacharya S, Butte AJ (2019). Prototype of running clinical trials in an untrustworthy environment using blockchain. Nat Commun.

[21] Omar IA, Jayaraman R, Salah K, Simsekler MCE, Yaqoob I, Ellahham S (2020). Ensuring protocol compliance and data transparency in clinical trials using blockchain smart contracts. BMC Med Res Methodol.

[22] Souza FS, Silva RPS, Junior LSB, Azevedo Filho HRC (2022). The evolution of neurosurgery throughout the ages: from trepanations in prehistory to the robotic era. Arquivos Brasileiros de Neurocirurgia: Brazilian Neurosurgery.

[23] Wu C-H, Liu C-Y (2022). Educational applications of non-fungible token (NFT). Sustainability.

[24] Blakeley JO, Ye X, Duda DG, Halpin CF, Bergner AL, Muzikansky A, Merker VL, Gerstner ER, Fayad LM, Ahlawat S, Jacobs MA, Jain RK, Zalewski C, Dombi E, Widemann BC, Plotkin SR (2016). Efficacy and biomarker study of bevacizumab for hearing loss resulting from neurofibromatosis type 2–associated vestibular schwannomas. J Clin Oncol.

[25] Gross AM, Glassberg B, Wolters PL, Dombi E, Baldwin A, Fisher MJ, Kim A, Bornhorst M, Weiss BD, Blakeley JO, Whitcomb P, Paul SM, Steinberg SM, Venzon DJ, Martin S, Carbonell A, Heisey K, Therrien J, Kapustina O, Dufek A, Derdak J, Smith MA, Widemann BC (2022). Selumetinib in children with neurofibromatosis type 1 and asymptomatic inoperable plexiform neurofibroma at risk for developing tumor-related morbidity. Neuro-Oncology.

[26] Stummer W, Pichlmeier U, Meinel T, Wiestler OD, Zanella F, Reulen H-J (2006). Fluorescence-guided surgery with 5-aminolevulinic acid for resection of malignant glioma: a randomised controlled multicentre phase III trial. Lancet Oncol.

[27] Chiacchio F, D’Urso D, Oliveri LM, Spitaleri A, Spampinato C, Giordano D (2022). A non-fungible token solution for the track and trace of pharmaceutical supply chain. Appl Sci.

[28] Durá M, Leal F, Sánchez-García Á, Sáez C, García-Gómez JM, Chis AE, González-Vélez H (2023) Blockchain for data originality in pharma manufacturing. J Pharm Innov. 10.1007/s12247-023-09748-z

[29] Samenjo KT, Oosting RM, Bakker C, Diehl JC (2023). The extent to which circular economy principles have been applied in the design of medical devices for low-resource settings in sub-Saharan Africa. A systematic review. Front Sustain.

[30] Shukla S, Kalaiselvan V, Singh Raghuvanshi R (2023). How to improve regulatory practices for refurbished medical devices. Bull World Health Organ.

[31] Gebreab SA, Salah K, Jayaraman R, Zemerly J (2023). Trusted traceability and certification of refurbished medical devices using dynamic composable NFTs. IEEE Access.

[32] Jean WC (2022). Virtual and augmented reality in neurosurgery: the evolution of its application and study designs. World Neurosurg.

[33] Pelargos PE, Nagasawa DT, Lagman C, Tenn S, Demos JV, Lee SJ, Bui TT, Barnette NE, Bhatt NS, Ung N, Bari A, Martin NA, Yang I (2017). Utilizing virtual and augmented reality for educational and clinical enhancements in neurosurgery. J Clin Neurosci.

[34] Cannavo A, Lamberti F (2021). How blockchain, virtual reality, and augmented reality are converging, and why. IEEE Consum Electron Mag.

[35] Beck R, Müller-Bloch C, King JL (2018) Governance in the blockchain economy: a framework and research agenda. J Assoc Inf Syst:1020–1034. 10.17705/1jais.00518

[36] Batko K, Ślęzak A (2022). The use of big data analytics in healthcare. J Big Data.

[37] Ofulue J, Benyoucef M (2022) Data monetization: insights from a technology-enabled literature review and research agenda. Manag Rev Q. 10.1007/s11301-022-00309-1

[38] Jung SY, Kim T, Hwang HJ, Hong K (2021). Mechanism design of health care blockchain system token economy: development study based on simulated real-world scenarios. J Med Internet Res.

[39] Esmaeilzadeh P (2023). Evolution of health information sharing between health care organizations: potential of nonfungible tokens. Interact J Med Res.

[40] Bamakan SMH, Nezhadsistani N, Bodaghi O, Qu Q (2022). Patents and intellectual property assets as non-fungible tokens; key technologies and challenges. Sci Rep.

[41] Aharon DY, Demir E (2022). NFTs and asset class spillovers: lessons from the period around the COVID-19 pandemic. Financ Res Lett.

[42] Enany S, Yaghy A, Alberto NRI, Alberto IRI, Bermea RS, Ristovska L, Yaghy M, Hoyek S, Patel NA, Celi LA (2023). The potential use of non-fungible tokens (NFTs) in healthcare and medical research. PLOS Digital Health.

[43] Lal A, You F (2023). Climate concerns and the future of nonfungible tokens: leveraging environmental benefits of the Ethereum merge. Proc Natl Acad Sci.

[44] Stoll C, Klaaßen L, Gallersdörfer U (2019). The carbon footprint of Bitcoin. Joule.

[45] Chalmers D, Fisch C, Matthews R, Quinn W, Recker J (2022). Beyond the bubble: will NFTs and digital proof of ownership empower creative industry entrepreneurs?. J Bus Ventur Insights.

[46] Bhujel S, Rahulamathavan Y (2022). A survey: security, transparency, and scalability issues of NFT’s and its marketplaces. Sensors.

[47] Ballantyne A (2020). How should we think about clinical data ownership?. J Med Ethics.

[48] Ali O, Momin M, Shrestha A, Das R, Alhajj F, Dwivedi YK (2023). A review of the key challenges of non-fungible tokens. Technol Forecast Soc Chang.

[49] Charles WM, Delgado BM (2022) Health datasets as assets: blockchain-based valuation and transaction methods. Blockchain in Healthcare Today. 10.30953/bhty.v5.185 10.30953/bhty.v5.185 PMC9907414 36779021

[50] Teo ZL, Ting DSW (2023). Non-fungible tokens for the management of health data. Nat Med.

